# Antitumor Study of the Miao Medicine *Indigofera stachyodes* by Integrating Multiple Chemometrics Network Pharmacology and Experimental Validation

**DOI:** 10.3390/cimb48030302

**Published:** 2026-03-12

**Authors:** Junhang Zhang, Dan Wang, Qin Nie, Huayong Lou, Yongping Zhang, Jian Xu, Jian Fu

**Affiliations:** 1 College of Pharmacy, Guizhou University of Traditional Chinese Medicine, Guiyang 550025, China; zjhzjh2026@126.com (J.Z.); 19184485123@163.com (D.W.); qnie2134@163.com (Q.N.); zgygpg@126.com (Y.Z.); 2 State Key Laboratory of Functions and Applications of Medicinal Plants, Guiyang 550014, China; loouhy@126.com; 3 National Engineering Technology Research Center for Miao Medicine, Guiyang 550025, China; 4 Guizhou Key Laboratory for Germplasm Innovation and Resource–Efficient Utilization of Dao-di Herbs, Guiyang 550025, China

**Keywords:** *Indigofera stachyodes*, fingerprints, antitumor, spectrum–effect relationship, network pharmacology, experimental validation

## Abstract

*Indigofera stachyodes* Lindl. (*I. stachyodes*), a fundamental herb in Miao ethnomedicine, possesses a broad pharmacological profile including antitumor potential. However, its antitumor bioactive compounds and their underlying mechanisms remain poorly characterized. Here, we developed a spectrum-effect relationship analysis integrated with UPLC-Q-TOF-MS/MS, which enabled the identification of 7 compounds with potential antitumor activity from *I. stachyodes*. A secondary screening of candidate compounds was performed using network pharmacology, which led to the identification of fisetin, luteolin, wogonin, and liquiritigenin as potential antitumor compounds. Enrichment analysis and molecular docking studies predicted the key involvement of the PI3K-AKT signaling pathway in mediating the antitumor activities of these compounds. Subsequently, in vitro cell experiments confirmed that the fisetin, wogonin, luteolin and liquiritigenin inhibited the proliferation of HepG2 cells, with IC_50_ values of 82.13 ± 6.74, 123.38 ± 5.71, 141.76 ± 6.37, and 151.04 ± 3.08 µM, respectively, while exhibiting moderate antitumor activity compared to chemotherapeutic agents. This antiproliferative effect was further corroborated by confocal laser scanning microscopy (CLSM). These results not only validate the potential of *I. stachyodes* as a source for antitumor agents but also provide a foundation for its further development.

## 1. Introduction

*Indigofera stachyodes* Lindl. (*I. stachyodes*) is predominantly distributed in southwestern China, including Guizhou, Yunnan, and Guangxi Provinces. Its root, known as “Xuerenshen” in Chinese, represents an important traditional medicine within the Miao ethnic group [1]. In traditional Chinese medicine (TCM), it is recognized for its ability to activate blood circulation (promote microcirculation and alleviate blood stasis), nourish yin (support body fluids and moisten tissues), eliminate phlegm, tonify the kidney, and promote diuresis [2]. Clinically, it has been used to treat conditions such as colds, fever, abdominal pain in women, liver cirrhosis, and rheumatic joint pain. Phytochemical investigations have revealed that *I. stachyodes* contains diverse bioactive compounds, such as flavonoids [3,4,5,6], tannins [7], glycosides, sterols [8], terpenes, phenolic acids, and volatile oils [9]. Our previous work reported that a series of novel compounds isolated from *I. stachyodes* exhibited remarkable pharmacological activities, including hepatoprotective [10,11], antibacterial [12], antitumor [13], anti-inflammatory [14,15,16], and antioxidant effects [17]. Notably, *I. stachyodes* serves as a key ingredient in QiJiaoShengBai (QJSB) capsules, a TCM formulation that has been clinically approved as an adjunctive therapy for tumors in China. QJSB has been shown to enhance the efficacy of chemotherapeutic agents and contribute to improved tumor control [18]. These findings collectively suggest its potential as a source of antitumor agents. However, although preliminary studies have suggested its antitumor potential, the relationship between the chemical constituents of this medicinal material and its bioactivity remains poorly understood. Specifically, the active substances responsible for its antitumor effects have yet to be elucidated, pharmacodynamics-related quality markers remain undefined, and the underlying molecular mechanisms lack systematic investigation. Moreover, existing quality control methods have not been correlated with antitumor activity, which hinders its standardized development and clinical application.

Fingerprint analysis, which provides a comprehensive chromatographic profile of characteristic components, has become a powerful tool for the quality assessment of complex herbal medicines [19]. When combined with chemometrics, it enables the correlation of chemical profiles with biological efficacy—an approach known as the spectrum-effect relationship. This strategy is crucial for identifying potential quality markers. Furthermore, given the multi-component, multi-target nature of TCM, network pharmacology offers a systematic framework for predicting drug-target interactions and elucidating complex mechanisms, aligning well with the holistic perspective of TCM [20]. Therefore, this study represents an initial effort to apply an integrated strategy combining spectrum-effect relationship analysis, network pharmacology, and in vitro experimental validation to investigate the antitumor potential of *I. stachyodes*, thereby contributing to filling a research gap for this medicinal material. The systematic application of this approach may help address the current lack of antitumor-associated quality markers for *I. stachyodes* and offers a scientific basis for the screening and prediction of its potential antitumor bioactive compounds.

This study aimed to systematically investigate the antitumor material basis of *I. stachyodes* and predict its potential mechanisms. First, UPLC fingerprints of 16 batches of *I. stachyodes* were established and analyzed using chemometric methods. The spectrum-effect relationship between fingerprint peaks and in vitro antitumor activity was then analyzed to screen potential active components, which were further identified by UPLC-Q-TOF-MS/MS. Subsequently, network pharmacology and molecular docking were employed to predict the key targets and pathways involved in the antitumor effects of these candidate compounds. Finally, the antiproliferative effects of *I. stachyodes* extracts and the identified active compounds were experimentally validated using HepG2 cells. Our findings are expected to provide a scientific basis for the quality control and further development of *I. stachyodes* as a potential antitumor TCM.

## 2. Materials and Methods

### 2.1. Establishment of Fingerprint Profiles and Evaluation of Pharmacological Activities of Indigofera stachyodes

#### 2.1.1. Medicinal Materials

All medicinal materials were collected from different regions of Guizhou Province, with detailed information provided in Table 1.

#### 2.1.2. Extraction of *Indigofera stachyodes*

Briefly, 100.0 g of *I. stachyodes* herb powder was first extracted 10 times (*v*/*w*, 1 L) with 90% (*v*/*v*) ethanol under reflux for 2 h, and the residue was extracted 10 times (*v*/*w*, 1 L) with 60% (*v*/*v*) ethanol for another 1 h. The combined filtrates were concentrated under reduced pressure at 50 °C until no ethanol odor was detectable, obtaining a concentrated extract. The concentrated *I. stachyodes* extract was freeze-dried (−40 °C, 130 Pa) and ground into powder.

#### 2.1.3. Sample Solution Preparation

A total of 0.5 g of the extract powder was accurately weighed and transferred into a 10 mL volumetric flask. Approximately 8 mL of methanol was added and sonicated for 10 min to dissolve the sample. The solution was allowed to cool to room temperature and then diluted to volume with methanol and mixed thoroughly. The solution was filtered through a 0.22 μm microporous membrane, and the resultant filtrate was used for injection.

#### 2.1.4. Preparation of Mixed Standard Solution

Approximately 5.0 mg of each reference compound was accurately weighed and transferred into separate 10 mL volumetric flasks. The standards were dissolved and diluted to volume with methanol to prepare individual stock solutions with a concentration of approximately 0.5 mg/mL. Then, 1.0 mL of each individual stock solution was precisely pipetted and combined into a single 10 mL volumetric flask. The mixture was diluted to volume with methanol to obtain the mixed reference standard solution.

#### 2.1.5. Chromatographic Condition

A Waters ACQUITY UPLC BEH Shield RP18 (2.1 mm × 100 mm, 1.7 μm, Waters Corporation, Milford, MA, USA) column was selected. Acetonitrile as the organic phase (C) and 0.1% phosphoric acid aqueous solution as the aqueous phase (A); gradient elution (0–5 min, 10–20% C; 5–25 min, 20–25% C; 25–31 min, 25–30% C; 31–46 min, 30–50% C; 46–50 min, 50–62% C); wavelength: 350 nm; temperature: 30 °C; flow rate: 0.2 mL/min; injection volume: 2 μL.

#### 2.1.6. Methodological Validation

Interassay precision was assessed by analyzing 5 injections of sample S1 solution. In addition, sample S1 was injected 5 times to evaluate its repeatability. Moreover, the stability was assessed by analyzing the sample S1 solution over a 24 h time period (0, 4, 8, 12, and 24 h).

#### 2.1.7. Establishment of Fingerprints and Similarity Evaluation

The UPLC fingerprints were generated via Origin software (version 2024). Common peaks and similarities were subsequently assessed with the “Similarity Evaluation System for Chromatographic Fingerprint of TCM (version 2012)”.

#### 2.1.8. UPLC-Q-TOF-MS/MS Analysis

UPLC-Q-TOF-MS/MS analysis was performed using a Waters Acquity UPLC HSS T3 (100 mm × 2.1 mm, 1.8 μm, Waters Corporation, Milford, MA, USA). The spray voltages were set to 3.0 kV in positive mode and 2.5 kV in negative mode. The capillary temperature was 320 °C. The mobile phases consisted of acetonitrile (A) and 0.1% aqueous formic acid (B) with a gradient elution of 5% A at 0–2 min, 5–95% A at 2–42 min, 95% A at 42–47 min, 5–95% B at 47–47.1 min and 95% B at 47.1–50 min. The flow rate was set at 0.3 mL/min, and the column temperature was maintained at 40 °C. The injection volume was 2 μL. The electrospray ionization source was set to positive and negative modes. Full scan mode was employed with a mass range of *m*/*z* 100–1500. MS data were collected using Thermo Xcalibur software (version 4.3).

#### 2.1.9. CCK-8 Cell Inhibition Assay

HepG2 cells were bought from Cell Bank, Chinese Academy of Sciences (Shanghai, China). Three types of control wells were set up: blank control (medium only, no cells), negative control (cells with medium), and experimental groups (cells with drug). The medium was DMEM high-glucose medium supplemented with 10% FBS. A total of 1 × 10^4^ HepG2 cells were seeded into a 96-well plate and cultured in DMEM medium containing different drug concentrations (final concentrations: 0, 25, 50, 100, 200, 400, 800, and 1600 µg/mL). After 24 h of incubation, 10 µL of CCK-8 reagent was added to each well, followed by further incubation at 37 °C for 3 h in the dark. Finally, the optical density (OD) at 450 nm was measured using a microplate reader, and the inhibition rate was calculated.

The inhibition rate (IR) of the HepG2 cells in each group was calculated via the following equation:

Inhibition Rate (%) = [1 − (OD450experimental − OD450blank)/(OD450control − OD450blank)] × 100%

#### 2.1.10. Validation of Antiproliferative Effect at a Selected Concentration

Based on the results from Section 2.1.9, the concentration of 100 μg/mL was found to be near the IC_50_ value and demonstrated significant inhibitory activity against HepG2 cells. Therefore, this concentration was selected for subsequent validation experiments. HepG2 cells were suspended in DMEM medium at a density of 1 × 10^4^ cells/mL and seeded into a 96-well plate. Each well was treated with different batches of *I. stachyodes* extract at a final concentration of 100 μg/mL. After 24 h of incubation, 10 μL of CCK-8 solution was added to each well, followed by an additional 3 h of culture in the dark. The optical density at 450 nm was measured using a microplate reader, and the inhibition rate was calculated accordingly.

#### 2.1.11. Statistical Analysis

Data were presented as the mean ± standard deviation (SD) of at least three independent experiments. Statistical significance was determined using one-way ANOVA followed by Tukey’s test for multiple comparisons against the negative control group. A *p*-value < 0.05 was considered statistically significant.

### 2.2. Investigation Based on Multiple Chemometric Methods

#### Chemometric Methods Analysis

To assess the overall differences in the chemical composition among different batches of *I. stachyodes* and to preliminarily explore its antitumor activity (along with potentially relevant chemical constituents), we conducted the following multivariate statistical analyses on the UPLC fingerprint data of all batch samples. Origin was used for hierarchical clustering analysis (HCA). Principal component analysis (PCA) and orthogonal partial least squares discrimination analysis (OPLS–DA) were conducted with SIMCA 14.1. MATLAB R2023a was used for gray relational analysis (GRA).

### 2.3. Network Pharmacology and Molecular Docking

#### 2.3.1. Target Prediction for *Indigofera stachyodes*

All identified or tentatively characterized compounds from *I. stachyodes* were subjected to ADME screening using the SwissADME database (http://www.swissadme.ch/, accessed on 5 January 2026). Compounds that met the following criteria were considered pharmaceutically active and selected for subsequent target prediction: GI Absorption (High) and drug-likeness (Lipinski’s rule of five violations ≤ 1). Potential targets of the compounds were identified using the Swiss Target Prediction database (http://www.swisstargetprediction.ch/, accessed on 5 January 2026), and those with a probability score exceeding 0.1 were selected for further investigation.

#### 2.3.2. Target Prediction for Tumors

Potential targets related to the tumor type were retrieved from the GeneCards database (https://www.genecards.org, accessed on 5 January 2026). Only genes with a score above or equal to the median value were retained for subsequent analyses, ultimately controlling the number of targets to between 1000 and 2000. The target set was complemented with data retrieved from the OMIM database (https://www.omim.org, accessed on 5 January 2026) and the DrugBank database (https://www.drugbank.com/, accessed on 5 January 2026).

#### 2.3.3. Construction of the “Drug and Target” Network

The active compounds and matching target network were constructed with Cytoscape 3.9.1.

#### 2.3.4. Construction of Protein–Protein Interactions Network

Targets were analyzed via the String database (https://string-db.org, accessed on 5 January 2026) to assess potential protein interactions (PPIs) between targets. The PPI network was constructed via Cytoscape 3.9.1.

#### 2.3.5. GO Function Analysis and KEGG Pathway Enrichment Analysis

The intersection targets were imported into the Metascape database (https://metascape.org, accessed on 5 January 2026) for GO analysis and KEGG enrichment analysis.

#### 2.3.6. Molecular Docking

The top targets identified from the PPI network (based on degree centrality) were selected as the core targets for molecular docking. Corresponding active compounds binding to these core targets were selected based on spectrum-effect relationship analysis. The files of the active compounds were retrieved from the PubChem database (https://pubchem.ncbi.nlm.nih.gov, accessed on 5 January 2026). The protein structure files of the core targets were obtained from the PDB database (https://www.rcsb.org/, accessed on 5 January 2026). Autodock (version 4.2.6) was used for molecular docking, and PyMol (version 3.1.7) was used for visualization.

### 2.4. Pharmacological Activity Validation of Monomeric Compounds

#### 2.4.1. In Vitro Experimental Validation

To validate the predictions from network pharmacology, four flavonoid compounds (liquiritigenin, fisetin, wogonin, and luteolin), which were identified as major constituents in chemical analysis, were selected for in vitro antitumor activity assessment. Liquiritigenin, fisetin, wogonin and luteolin were dissolved in DMSO and diluted to different concentrations with DMEM medium (the final concentration of DMSO was 0.1%). A total of 1 × 10^4^ HepG2 cells were inoculated in 96–well plates, and the cells were treated with different concentrations of compounds (25, 50, 100, 200, and 400 µM) for 24 h, with six replicates per concentration, and each experiment was independently repeated three times. After treatment with the compounds for 24 h, 10 μL of CCK-8 solution was added to each well, and the plates were further incubated for 3 h at 37 °C in the dark. After incubation, the optical density (OD) at 450 nm was measured with a microplate reader and the cell inhibition rate was calculated.

#### 2.4.2. DAPI/PI Fluorescence Staining

To assess the induction of cell death by the active compounds, HepG2 cells were seeded in 24-well plates at a density of 1 × 10^4^ cells per well. After attachment, cells were treated with liquiritigenin, fisetin, wogonin, and luteolin at their respective 2 × IC_50_ concentrations for 24 h. After treatment, the medium was aspirated, and cells were washed gently twice with PBS. Subsequently, 20 µL of DAPI (10 µg/mL) and 20 µL of PI (20 µg/mL) were added, followed by incubation for 30 min in the dark. Observations and imaging were performed via confocal laser scanning microscopy (200×). For quantitative analysis, three random fields were captured per well. Using ImageJ software (version 1.54), the number of total nuclei (DAPI-positive) and PI-positive nuclei were counted. The PI-positive cells rate (%) was calculated as (Number of PI-positive nuclei/Number of total nuclei) × 100%. Data from three independent experiments were pooled for statistical analysis.

## 3. Results

### 3.1. UPLC Fingerprints and UPLC-Q-TOF-MS/MS Analysis

#### 3.1.1. Methodological Validation

The results of this part are shown in the supporting information (Figures S1–S3).

#### 3.1.2. UPLC Fingerprints

**Figure 1:**
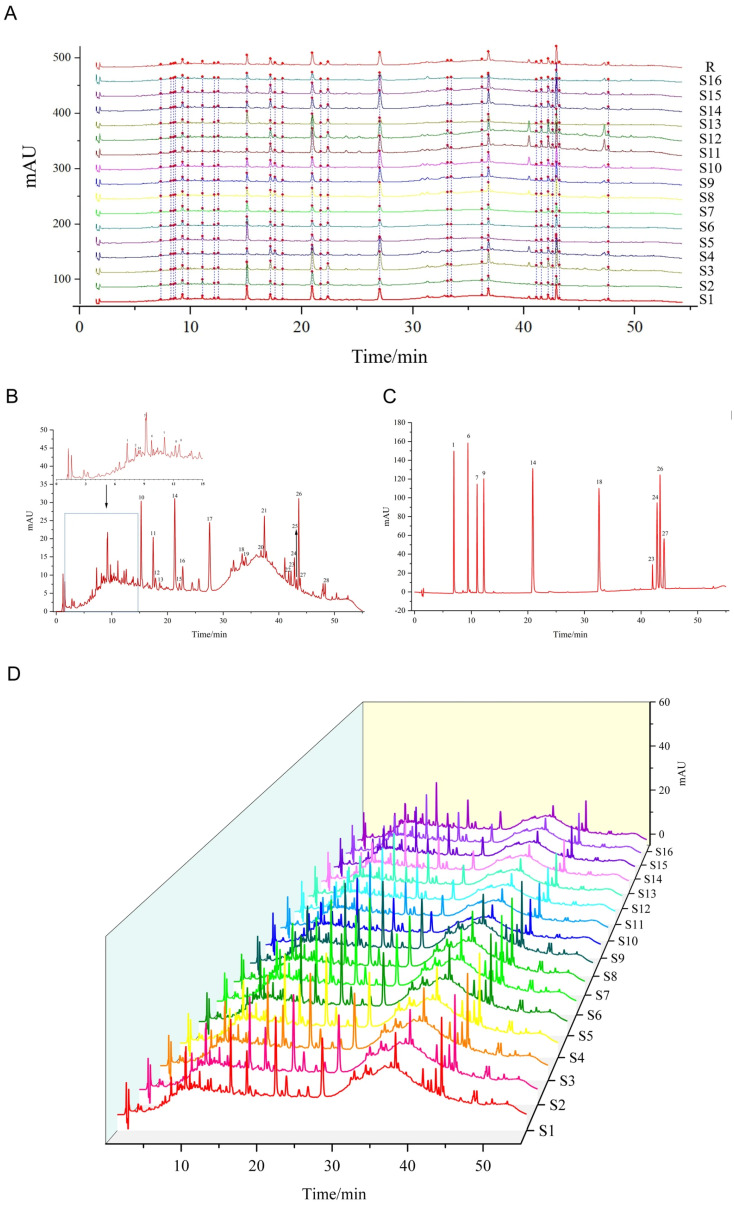
(**A**) Common peaks of *Indigofera stachyodes*. (**B**) UPLC of S1 sample. (**C**) Mixed reference substances. (**D**) UPLC fingerprints of 16 batches of *Indigofera stachyodes*.

Twenty-eight common peaks were obtained from the analysis of the UPLC fingerprints (Figure 1A,D). The similarity results are illustrated in Table S1, which presents favorable similarity of *I. stachyodes* across all groups. By comparing with the reference substances (epicatechin, vitexin, luteolin-7-*O*-glucoside, hyperoside, fisetin, luteolin, glycyrrhizic acid, wogonin, liquiritigenin, and isoliquiritigenin) as shown in Figure 1B,C, ten common peaks were identified: peak 1 was epicatechin, peak 6 was vitexin, peak 7 was luteolin-7-*O*-glucoside, peak 9 was hyperoside, peak 14 was fisetin, peak 18 was luteolin, peak 23 was glycyrrhizic acid, peak 24 was wogonin, peak 26 was liquiritigenin, and peak 27 was isoliquiritigenin.

#### 3.1.3. UPLC-Q-TOF-MS/MS Analysis

The total ion current chromatogram of *I. stachyodes* is shown in Figure 2. The identified compounds were verified by UPLC-Q-TOF-MS/MS (Figures S4–S10). Seven compounds were successfully validated, as shown in Table 2.

### 3.2. Antitumor Activity of Indigofera stachyodes

#### 3.2.1. Cell Inhibition

**Figure 3:**
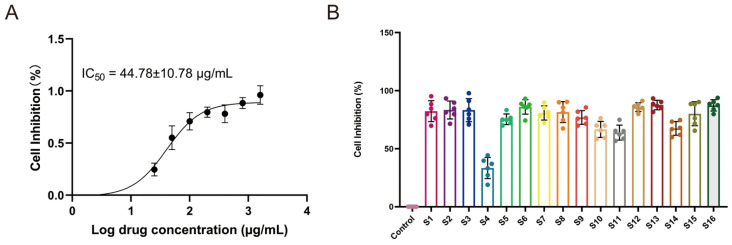
(**A**) Results of CCK-8 analysis (mean ± SD, *n* = 6). (**B**) Inhibition of *Indigofera stachyodes* in HepG2 cells (*n* = 6).

As shown in Figure 3A, 25–1600 μg/mL extract clearly inhibited the growth of HepG2 cells in a dose-dependent manner. The IC_50_ value of *I. stachyodes* for HepG2 cells was 44.78 ± 10.78 µg/mL.

#### 3.2.2. Validation of Antiproliferative Effect at a Selected Concentration

As shown in Figure 3B, all groups except S4 significantly inhibited tumor cell growth compared to the control group (Table S2). The scatter points within each group were evenly distributed with low intra-group variability, indicating stable pharmacological efficacy of *I. stachyodes* across treatments.

### 3.3. Spectrum–Efficacy Relationship Results

#### 3.3.1. Hierarchical Cluster Analysis (HCA)

**Figure 4:**
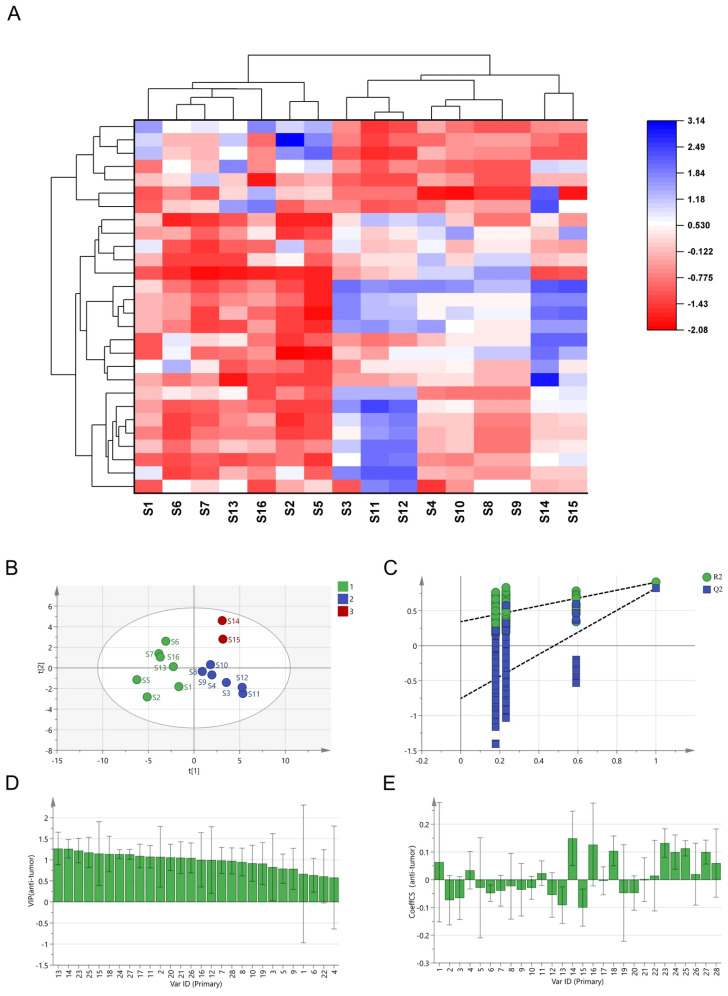
(**A**) Results of HCA. (**B**) Results of PCA. (**C**) Permutation test (*n* = 200). (**D**) VIP contribution plot of common peaks of potential antitumor components. (**E**) Correlation coefficients of antitumor activity.

Z-score normalization was applied to the peak areas of 28 common peaks, followed by hierarchical clustering using the squared Euclidean distance as the clustering criterion. The cluster heatmap was then generated using Origin. As illustrated in the heat map (Figure 4A), the samples were segregated into three distinct clusters: Group A (S1, S2, S5, S6, S7, S13, S16), Group B (S3, S4, S8, S9, S10, S11, S12), and Group C (S14, S15).

#### 3.3.2. Principal Component Analysis (PCA)

The peak areas after Z-score normalization were subjected to PCA using SIMCA 14.1. The model exhibited R^2^ = 0.773 and Q^2^ = 0.510, both exceeding the commonly accepted threshold of 0.5, indicating adequate explanatory and predictive capability for the dataset. As illustrated in Figure 4B, the three groups of *I. stachyodes* samples exhibited a separation trend in the analysis, indicating differences in their chemical composition. To precisely identify the differential components among the groups, OPLS-DA was employed for modeling. The obtained model parameters were R^2^X = 0.774, R^2^Y = 0.861, and Q^2^ = 0.718. The risk of overfitting was evaluated by a permutation test (*n* = 200). As shown in Figure S11, the regression line of the permuted Q2 values intersected the vertical axis (Y-axis) at a negative value. According to established criteria, these results confirm that the original OPLS-DA model was valid, and not overfitted. Based on the criterion of variable importance in projection (VIP) values greater than 1.0 (Figure S12), 14 peaks were identified as major contributors to the separation among the three groups in the OPLS-DA model. These included peaks such as liquiritigenin (peak 26), hyperoside (peak 9), luteolin-7-O-glucoside (peak 7), and wogonin (peak 24). To our knowledge, luteolin-7-O-glucoside and wogonin have not been previously reported as potential candidate markers for distinguishing the quality variations in *I. stachyodes* in the literature.

#### 3.3.3. Orthogonal Partial Least Squares Discriminant Analysis (OPLS–DA)

In the OPLS–DA model, the common peak areas were defined as the independent variables, and the tumor inhibition rate was selected as the dependent variable. The regression coefficient and the VIP score results were obtained from Simca 14.1. The established model parameters R^2^X, R^2^Y, and Q^2^ were 0.766, 0.929 and 0.813, respectively, all of which approached 1 [21], indicating that the model had an excellent fit and a strong predictive ability for the observed antitumor activity data within this sample set. The permutation test was carried out on the established model (Figure 4C). The validity of the model was assessed by a permutation test (*n* = 200, Figure 4C). In the permutation plot, all permuted models (blue dots) yielded lower R^2^Y and Q^2^ values compared to the original model (green dot). Furthermore, the regression line of the permuted Q^2^ values intersected the Y-axis at a negative value. These outcomes confirm that the original model was robust and not overfitted. According to the principle of a VIP value greater than 1 [22], 14 compounds were identified as potential antitumor components. The VIP values were ranked in the order of peak 13 > peak 14 > peak 23 > peak 25 > peak 15 > peak 18 > peak 24 > peak 27 > peak 17 > peak 11 > peak 2 > peak 20 > peak 21 > peak 26 (Figure 4D). Figure 4E clearly shows that peaks 1, 4, 11, 14, 16, 18, 21, 22, 23, 24, 25, 26, and 27 have appropriate positive correlation coefficients. In other words, the pharmacologically active substances of *I. stachyodes* contributing to its antitumor effects may include peaks 11, 14 (fisetin), 18 (luteolin), 21, 23 (glycyrrhizic acid), 24 (wogonin), 25, 26 (liquiritigenin), and 27 (isoliquiritigenin).

#### 3.3.4. Gray Relational Analysis (GRA)

Gray relational analysis (GRA) was performed according to the method described in Section 3.3.3, and the calculated gray relational grades (GRGs) for each common peak are listed in Table 3. The GRGs ranged from 0.5997 to 0.8336. Using a threshold of GRG > 0.70 to indicate a strong association, 7 peaks were identified. These GRG-based results were largely consistent with those from the OPLS-DA model (VIP > 1.0). The convergence of results from OPLS-DA and GRA strengthens the evidence that peaks 14, 18, 24, 25, and 26 are key components associated with the antitumor efficacy of *I. stachyodes*.

### 3.4. Network Pharmacology

#### 3.4.1. Active Compounds and Potential Targets of *Indigofera stachyodes*

We retrieved 33 active compounds (Table 4) and 713 potential targets from *I. stachyodes*. After removing duplicates, two hundred potential targets related to the 33 compounds were identified.

#### 3.4.2. Screening of Disease Targets

**Figure 5:**
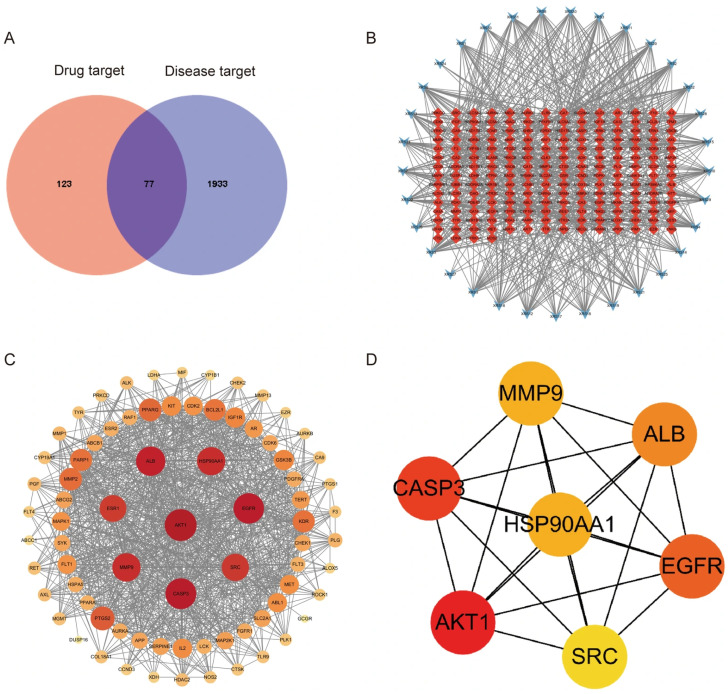
(**A**) Venn diagram. (**B**) “Active compounds and corresponding targets” network. (**C**) PPI network. (**D**) Core targets.

In this study, we obtained 2010 antitumor targets from the Gene Card database, supplemented with the OMIM database and the DrugBank database. The drug targets were intersected with disease targets via bioinformatics, and 77 drug-disease targets emerged (Figure 5A).

#### 3.4.3. Network of Compounds and Targets

The network of active compounds and corresponding targets is displayed in Figure 5B. The analysis revealed that multiple active compounds interact with several targets, highlighting the multi-target nature of *I. stachyodes*.

#### 3.4.4. Construction of a Potential Protein Interaction Network

The PPI network was constructed based on the 77 intersection targets from the String database and visualized using Cytoscape 3.9.1 (Figure 5C). Figure 5D displays the core targets screened by CytoHubba.

#### 3.4.5. GO and KEGG Analysis

**Figure 6:**
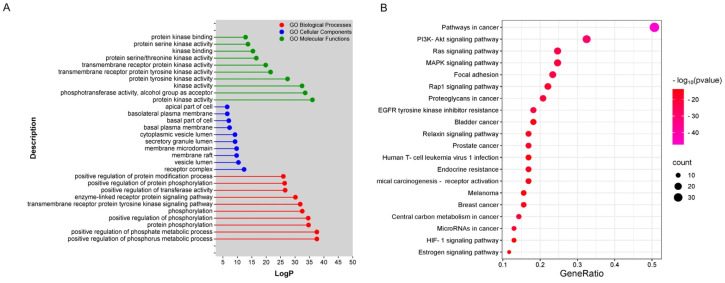
(**A**) GO and (**B**) KEGG analysis.

Gene Ontology (GO) annotation and Kyoto Encyclopedia of Genes and Genomes (KEGG) analysis were carried out on intersecting targets via the Metascape database. The top 10 GO terms were plotted according to their logP values (Figure 6). According to the GO biological process (BP) results, the targets were strongly involved in positive regulation of phosphorus metabolism, positive regulation of phosphate metabolism, protein phosphorylation, etc. The GO cell composition (CC) terms included the receptor complex, vesicle lumen, membrane raft, etc. The GO molecular function (MF) terms were related to protein kinase activity, kinase activity, phosphotransferase activity of alcohol--based receptors, etc. The enrichment analysis of the KEGG signaling pathways included 156 terms, which suggested that the antitumor action of *I. stachyodes* mainly involved the PI3K−Akt signaling pathway, Ras signaling pathway, MAPK signaling pathway, focal adhesion, Rap1 signaling pathway and others. The top 20 pathways according to the gene ratio are displayed in a bubble chart (Figure 6).

#### 3.4.6. Molecular Docking

**Figure 7:**
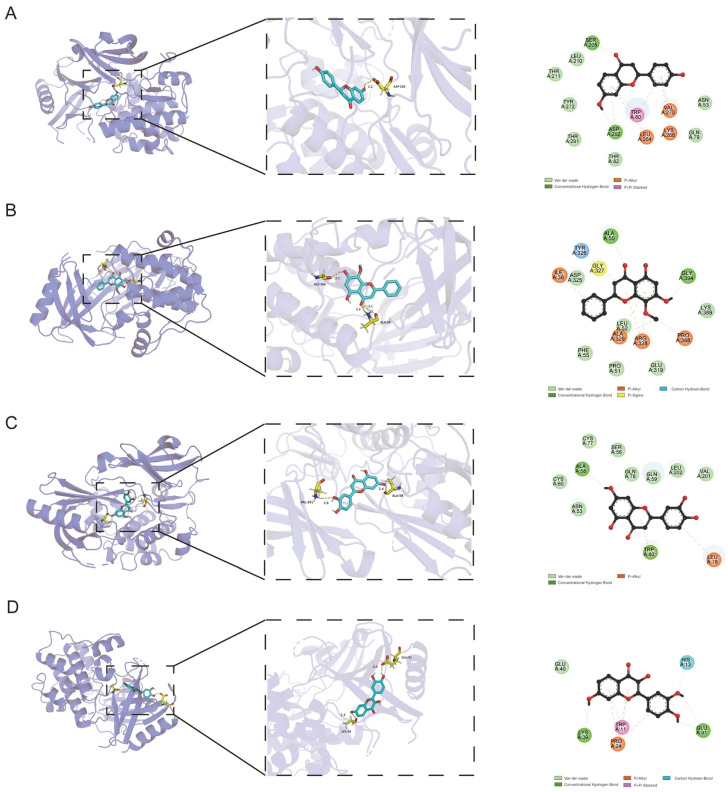
Predicted binding mode of ligands and AKT1. (**A**) Liquiritigenin, (**B**) Wogonin, (**C**) Luteolin, and (**D**) Fisetin, demonstrating key ligand--binding residues in the active site.

The core targets of AKT1 were docked with fisetin, luteolin, wogonin, and liquiritigenin. The binding energies are shown in Table 5. All compounds displayed observable affinity for AKT1, despite having predicted binding energies marginally lower than the positive control, MK-2206. The molecular simulations were visualized with PyMol (Figure 7A–D).

### 3.5. In Vitro Experimental Validation

#### 3.5.1. Inhibition of HepG2 Cell Proliferation by the Four Compounds

**Figure 8:**
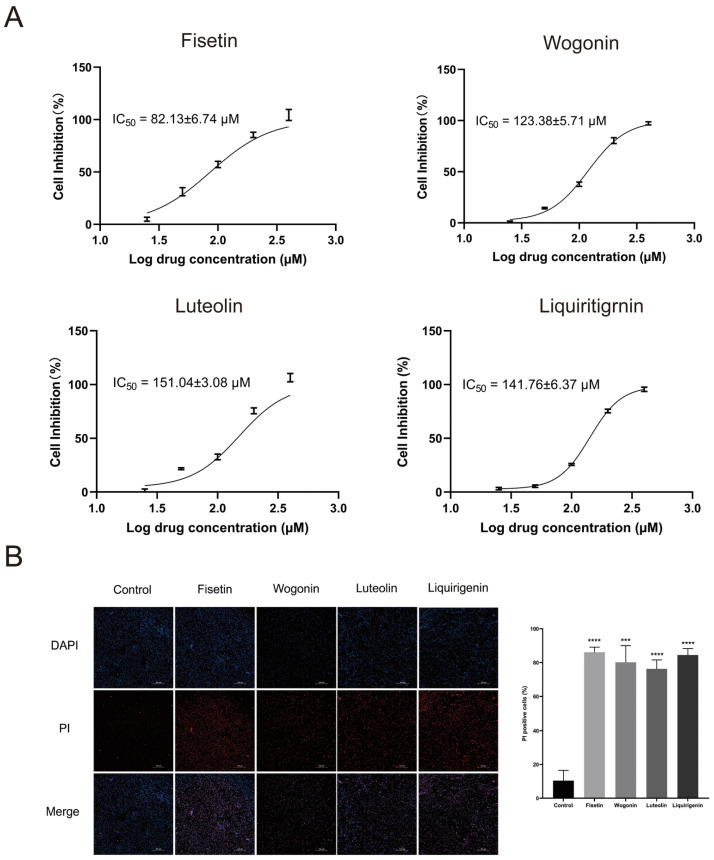
(**A**) Results of CCK-8 analysis and IC_50_. (**B**) Fluorescence micrographs of HepG2 cells stained with DAPI, PI (200×) and statistical analysis of cell viability by PI staining. Fisetin vs. control, **** *p* < 0.0001. Wogonin vs. control, *** *p* <0.0005. Luteolin vs. control, **** *p* < 0.0001. Liquiritigenin vs. control, **** *p* < 0.0001.

Nonlinear fitting of the data with GraphPad Prism 9.5 (Figure 8A) demonstrated that fisetin, wogonin, liquiritigenin, and luteolin all exhibited significant antitumor activity, with IC_50_ values of 82.13 ± 6.74, 123.38 ± 5.71, 141.76 ± 6.37 and 151.04 ± 3.08 µM, respectively. To enable a comparison of in vitro activity on an equivalent mass concentration basis, the IC_50_ values of the four compounds were converted from molar concentration (μM) to mass concentration (μg/mL) using the formula: mass concentration = molar concentration × molecular weight. The results showed that the IC_50_ values of fisetin, wogonin, liquiritigenin, and luteolin were 23.51 ± 1.93, 35.05 ± 1.62, 36.30 ± 1.63, and 43.23 ± 0.88 μg/mL, respectively.

#### 3.5.2. DAPI/PI Fluorescence Staining

As shown in Figure 8B, the majority of cells in the blank control group exhibited blue nuclear fluorescence with DAPI staining and maintained an intact morphology. In contrast, HepG2 cells treated with the 4 monomeric compounds displayed distinct propidium iodide (PI) red fluorescence in addition to DAPI blue fluorescence. PI is a nucleic acid dye that can only enter the nucleus and bind to DNA when the cell membrane integrity is compromised. Therefore, the presence of PI--positive signals indicates increased membrane permeability and loss of membrane integrity, which are characteristic features of cell death. Quantitative analysis of PI staining (using ImageJ software) demonstrated that all four compounds significantly increased the percentage of PI-positive HepG2 cells (*p* < 0.01). The finding that these compounds compromised HepG2 cell membrane integrity provides a mechanistic basis for their potent growth-inhibitory effect. Furthermore, direct morphological analysis confirmed that all four compounds induced cell death, thereby demonstrating a key pathway through which they exert their antitumor activity.

## 4. Discussion

In this study, we established a UPLC fingerprint for *I. stachyodes* and developed a spectrum-effect relationship model to investigate its antitumor properties. The identification of luteolin-7-glucoside and wogonin, reported here for the first time in this plant, enriches its known phytochemical profile. By integrating chemometric models (OPLS-DA, GRA) with network pharmacology, we adopted a strategy that not only identifies efficacy-correlated markers but also proposes potential mechanisms. This integrated analysis converged on four flavonoids—fisetin, luteolin, wogonin, and liquiritigenin—as the key compounds potentially responsible for the antitumor effect of *I. stachyodes*.

The selection of these four compounds is supported by both our predictive models and existing literature. Fisetin, luteolin, wogonin, and liquiritigenin have all been reported to possess antitumor activities, with a notable convergence on the PI3K-Akt signaling pathway as a common target. Fisetin induces apoptosis through ROS generation and by suppressing the PI3K-Akt, NF-κB, and MAPK pathways [23,24]. Luteolin inhibits the PI3K-Akt and NF-κB pathways, activating p53-dependent apoptosis [25]. Wogonin directly binds to PI3K, blocking Akt phosphorylation [26,27]. Liquiritigenin induces autophagy by inhibiting the mTOR pathway downstream of PI3K-Akt [28]. Based on the aforementioned literature, we speculate that these compounds synergistically modulate the PI3K-Akt pathway, a finding that is in line with our network pharmacology predictions.

Our network pharmacology and KEGG enrichment analysis further substantiated this focus. The antitumor action of *I. stachyodes* was predicted to primarily involve the PI3K-Akt, Ras, and MAPK signaling pathways, with the PI3K-Akt pathway emerging as the most significantly enriched pathway and the primary target of the identified compounds, as evidenced by its high -Log10(P) and GeneRatio. Molecular docking studies predicted AKT1 as a core target, with fisetin, luteolin, wogonin, and liquiritigenin all demonstrating favorable binding affinities. As a central node in the PI3K-Akt pathway, AKT1 promotes tumor survival and growth through multiple mechanisms, including mTOR activation and inhibition of pro-apoptotic signals [29,30]. Therefore, we hypothesize that the antitumor effect of *I. stachyodes* may be mediated, at least in part, through these key flavonoids concurrently modulating the PI3K-Akt pathway via interactions with targets like AKT1. The CCK-8 assay revealed that the IC_50_ values of the individual compounds ranged from 23.51 to 43.23 μg/mL, while that of the crude extract was 44.78 μg/mL. This comparison suggests that the extract may exhibit greater antiproliferative potency than the individual compounds, considering that the extract contains other constituents. However, this experiment has certain limitations, as the respective contributions of these compounds to the overall antitumor activity cannot be determined at this stage. Future studies will employ UPLC quantification to determine the content of these compounds in the extract, thereby enabling a more precise assessment of their individual contributions. The variation in potency among these structurally related flavonoids may be attributed to differences in cellular uptake, metabolic stability, or precise interactions with their molecular targets, which warrants further investigation [31]. Furthermore, CLSM imaging following PI staining revealed distinct red fluorescence in treated cells, indicating a loss of membrane integrity consistent with late apoptosis or necrosis [32]. Quantitative analysis of PI staining corroborated this visual evidence. These results provide direct in vitro confirmation of the antiproliferative and cell-death-inducing effects of the four candidate compounds, thereby lending experimental support to the predictions derived from our spectrum-effect and network pharmacology models.

This study has several limitations that should be addressed in future work. The PCA and OPLS-DA models were developed using a limited number of samples and variables, which poses a risk of overfitting. While permutation tests play a pivotal role in confirming the models’ internal validity, the absence of an independent external validation set limits our ability to assess their predictive power and generalizability. Consequently, these results should be regarded as preliminary, and further validation with larger, independent cohorts is essential to establish the robustness and applicability of our findings. The in vitro pharmacological evaluation in this study was conducted exclusively using the HepG2 human hepatocellular carcinoma cell line. Although HepG2 is a well-established and widely used model in liver cancer research, the use of a single cell line limits the generalizability of the findings. Future studies should include additional representative liver cancer cell lines to validate the broad-spectrum antitumor activity of *I. stachyodes*. Although network pharmacology and molecular docking predicted the involvement of the PI3K-Akt pathway and the AKT1 target, this study did not validate whether these compounds modulate this pathway at the protein level, nor did it elucidate the underlying molecular mechanisms. Notably, the PI3K-Akt pathway is central to the overall narrative of this study, as it was not only the most statistically significant pathway in the KEGG enrichment analysis but also a common node previously reported for all four compounds. This lack of validation limits, to some extent, the depth of the conclusions. The current data only demonstrate that the four compounds inhibit the proliferation of HepG2 cells but do not directly establish that these effects are mediated via the PI3K-Akt pathway. Future studies should employ protein analysis techniques to assess changes in AKT phosphorylation levels and the expression of its downstream effectors following treatment with the four compounds, thereby verifying the involvement of this pathway. As a database-dependent computational prediction approach, network pharmacology has inherent limitations that warrant objective consideration in the present study. First, network pharmacology relies heavily on the completeness and accuracy of existing bioinformatics databases. Second, the chemical composition of natural products is highly complex and diverse, and current databases typically only include components that are present in high abundance or have been extensively studied. Many trace constituents with significant bioactivity, as well as in vivo metabolites of the parent compounds, are often overlooked, potentially leading to the omission of key pharmacologically active substances. In summary, the primary value of network pharmacology at its current stage lies in providing research clues and generating scientific hypotheses, rather than directly revealing biological truths. Therefore, the prediction results obtained through network pharmacology in this study should be regarded as exploratory findings, which require subsequent systematic experimental validation to confirm their scientific significance [33,34]. Furthermore, this study has certain limitations in the dose–response analysis, as the morphological characteristics of the dose–effect curves were not investigated in depth. In addition, the analysis of potential synergistic or antagonistic interactions among the different compounds, as well as the multi-target mechanisms, remains primarily based on network pharmacology predictions and lacks corresponding pharmacological experimental validation. Future studies should employ systematic combination assays and target-specific functional analyses to further elucidate the synergistic mechanisms and multi-target regulatory networks underlying the antitumor effects of these compounds.

## 5. Conclusions

In summary, by employing an integrated strategy of chemical fingerprinting, spectrum-effect relationship modeling, and network pharmacology, this study suggests that fisetin, luteolin, wogonin, and liquiritigenin are potential key flavonoids associated with the antitumor property of *I. stachyodes*. Subsequent in vitro experiments demonstrated their antiproliferative activity against HepG2 cells, thereby providing preliminary experimental support for the predictions generated by the computational models. This work not only enriched the phytochemical profile of *I. stachyodes* but also provided preliminary insights into the chemical basis and potential mechanisms underlying the antitumor application of this ethnic medicine, highlighting its promise as a candidate for further preclinical development.

## Figures and Tables

**Figure 2:**
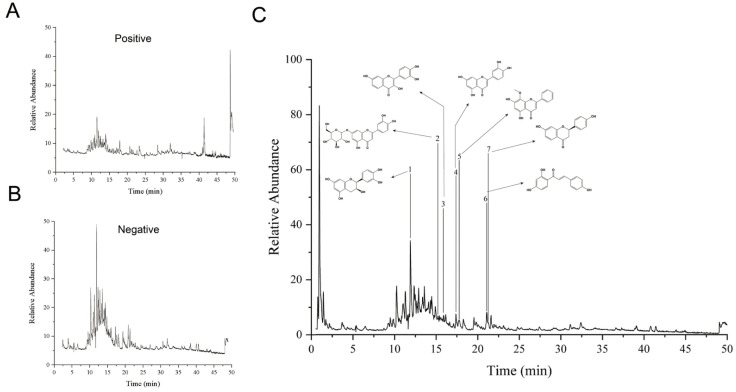
(**A**) Positive ion mode. (**B**) Negative ion mode. (**C**) Total ion chromatogram of UPLC-Q-TOF-MS/MS of *Indigofera stachyodes*.

**Table 1:** Information of medicinal materials.

No.	Origin of Medicinal Materials
S1	Guiding county in Guizhou province, China
S2	Xiuwen county in Guizhou province, China
S3	Wudang District in Guizhou province, China
S4	Dejiang county in Guizhou province, China
S5	Libo county in Guizhou province, China
S6	Dushan county in Guizhou province, China
S7	Longli county in Guizhou province, China
S8	Yuqing county in Guizhou province, China
S9	Meitan county in Guizhou province, China
S10	Bozhou District in Guizhou province, China
S11	Pingba District in Guizhou province, China
S12	Kaiyang county in Guizhou province, China
S13	Panzhou City in Guizhou province, China
S14	Huaxi District in Guizhou province, China
S15	Xishui county in Guizhou province, China
S16	Liuzhi Special District in Guizhou province, China

**Table 2:** Results of UPLC-Q-TOF-MS/MS analysis.

No.	Retention Time	Theoretical *m*/*z*	Measured *m*/*z*	Molecular Formula	MS/MS Fragments	Compound
1	11.95	290.0790	290.0710	C_15_H_14_O_7_	245.0811 137.0228 109.0280	Epicatechin
2	15.38	448.1005	448.0921	C_21_H_20_O_11_	285.0396 257.0448 151.0023	Luteolin 7-*O*-glucoside
3	16.14	286.0477	286.0396	C_15_H_10_O_6_	257.0456 135.0073 121.0280	Fisetin
4	17.36	286.0477	286.0758	C_15_H_10_O_6_	257.0456 239.0344 151.0392	Luteolin
5	17.81	284.0684	284.0759	C_16_H_12_O_5_	285.0759 270.0522 253.0494	Wogonin
6	21.66	256.0735	256.0654	C_15_H_12_O_4_	153.0178 135.0073 119.0488	Liquiritigenin
7	21.88	256.0735	256.0654	C_15_H_12_O_4_	153.0178 135.0073 119.0487	Isoliquiritigenin

**Table 3:** The results of GRA.

No.	GRG	No.	GRG
P1	0.6801	P15	0.7121
P2	0.7432	P16	0.6875
P3	0.6690	P17	0.6267
P4	0.7604	P18	0.7074
P5	0.8064	P19	0.6961
P6	0.6939	P20	0.6759
P7	0.7170	P21	0.6560
P8	0.7888	P22	0.7155
P9	0.7237	P23	0.7247
P10	0.7108	P24	0.7752
P11	0.6178	P25	0.7052
P12	0.7016	P26	0.7857
P13	0.6790	P27	0.5997
P14	0.7799	P28	0.8336

**Table 4:** Active compounds of *Indigofera stachyodes*.

No.	Compounds Name
XRS1	7,3′,5′-Trihydroxyflavanone
XRS2	7-Hydroxyl-4′-methoxyflayanone
XRS3	Liquiritigenin
XRS4	3′-Methoxydaidzein
XRS5	Calycosin
XRS6	Formononetin
XRS7	Daidzein
XRS8	Catechin
XRS9	Farnisin
XRS10	Garbanzol
XRS11	Luteolin
XRS12	3-(3,4-Dimethoxyphenyl)-3,4-dihydro-2H-chromen-7-ol
XRS13	Genistein
XRS14	Maackiain
XRS15	Fisetin
XRS16	(2Z)-6-Hydroxy-2-[(3-hydroxy-4-methoxyphenyl)methylidene]-1-benzofuran-3-one
XRS17	Sulfuretin
XRS18	7-Hydroxy-4-benzopyrone
XRS19	5,7-Dihydroxychromone
XRS20	Wogonin
XRS21	Sativan
XRS22	9-Methoxy-6a,11a-dihydro-6H-[1]benzofuro [3,2-c]chromen-3-ol
XRS23	2′,4′-Dihydroxychalcone
XRS24	(+) Stachyols A
XRS25	(−) Stachyols B
XRS26	(−) Stachyols A
XRS27	(+) Stachyols B
XRS28	Stachyols C
XRS29	Stachyols D
XRS30	Protocatechuic acid
XRS31	Gallic acid
XRS32	Gentisic acid
XRS33	Protocatechualdehyde

**Table 5:** Molecular binding energy.

Compounds	Target	Binding Energy (Kcal/mol)
Liquiritigenin	AKT1	−4.87
Wogonin	AKT1	−4.44
Luteolin	AKT1	−3.25
Fisetin	AKT1	−3.12
MK-2206	AKT1	−6.13

## Data Availability

The original contributions presented in this study are included in the article/Supplementary Material. Further inquiries can be directed to the corresponding authors.

## Supplementary Materials

The following supporting information can be downloaded at: https://www.mdpi.com/article/10.3390/cimb48030302/s1.

## Abbreviations

The following abbreviations are used in this manuscript:

TCM	Traditional Chinese Medicine
PCA	Principal component analysis
HCA	Hierarchical cluster analysis
GRA	Gray relational analysis
OPLS-DA	Orthogonal partial least squares discriminant analysis
PPI	Protein–protein interaction
GO	Gene Ontology
KEGG	Kyoto Encyclopedia of Genes and Genomes
AKT1	AKT serine/threonine kinase 1
