# Supplementary material for: Antitumor Study of the Miao Medicine Indigofera stachyodes by Integrating Multiple Chemometrics Network Pharmacology and Experimental Validation
Source: Curr Issues Mol Biol. 2026 Mar 12;48(3):302. doi: 10.3390/cimb48030302 (PMC13025688; doi:10.3390/cimb48030302)
Supplement: Supplementary file 1 [file cimb-48-00302-s001.zip › cimb-4174672-supplementary.pdf]

current issues in molecular biology

Article

Antitumor Study of the Miao Medicine *Indigofera stachyodes* by Integrating Multiple Chemometrics Network Pharmacology and Experimental Validation

Junhang Zhang, Dan Wang, Qin Nie, Huayong Lou, Yongping Zhang, Jian Xu* and Jian Fu*

Supplement Materials

1. *Methodological validation*

1.1. Precision

The precision of the assay was evaluated by performing 5 consecutive injections of a mixed reference standard. The results demonstrated that the relative standard deviation (RSD) of the relative peak areas for the common peaks fell within the range of 0.56% to 1.00%.

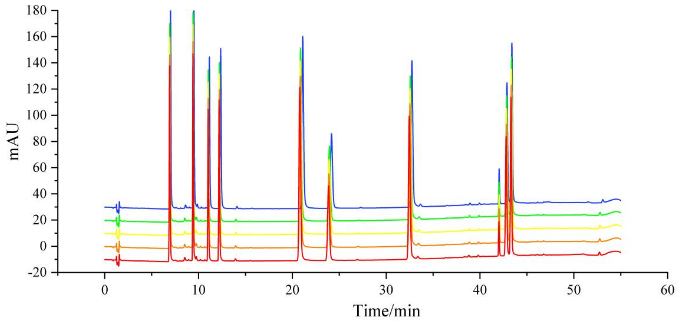

Figure S1. Chromatograms for precision evaluation

1.2. Repeatability

The repeatability was assessed by performing five consecutive injections of the sample S1 solution. The RSD for the relative peak areas of common peaks ranged from 0.26% to 2.49%.

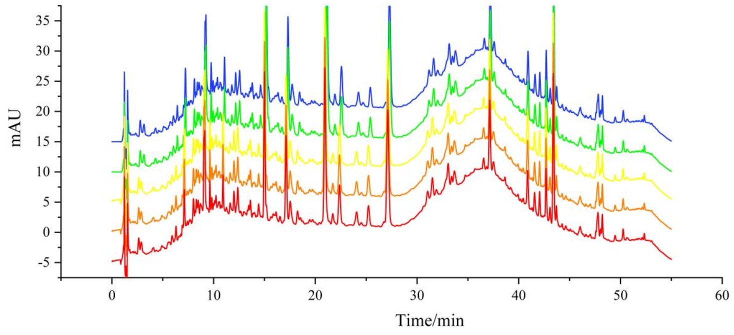

Figure S2. Chromatograms for repeatability evaluation

1.3. Stability

The stability profile of sample S1 solution, analyzed over a 24-hour period at specified time points (0, 4, 8, 12, and 24 h), showed RSD of 0.20% to 1.70% for the relative peak areas of common peaks.

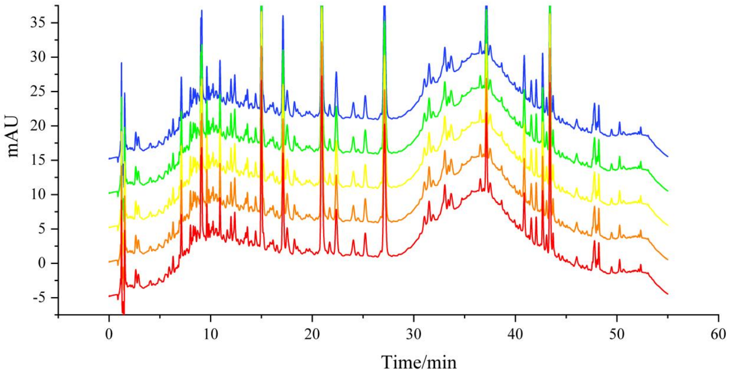

Figure S3. Chromatograms for stability evaluation

2. *Similarity of 16 Indigofera stachyodes*

Table S1. Similarity of 16 *Indigofera stachyodes*

No.	Similarity	No.	Similarity
S1	0.981	S9	0.984
S2	0.937	S10	0.881
S3	0.985	S11	0.898
S4	0.879	S12	0.973
S5	0.973	S13	0.944
S6	0.951	S14	0.982
S7	0.949	S15	0.968
S8	0.980	S16	0.991

3. *UPLC-Q-TOF-MS/MS results*

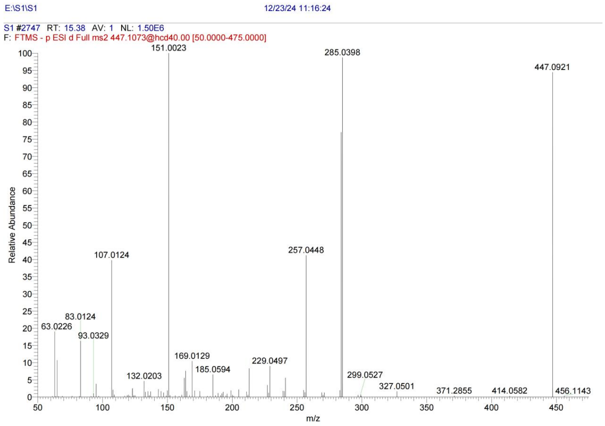

Figure S4. Luteolin-7-O-glucoside MS/MS fragments

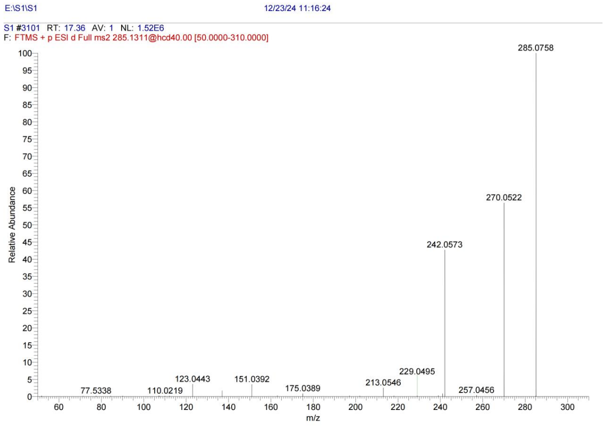

Figure S5. Luteolin MS/MS fragments

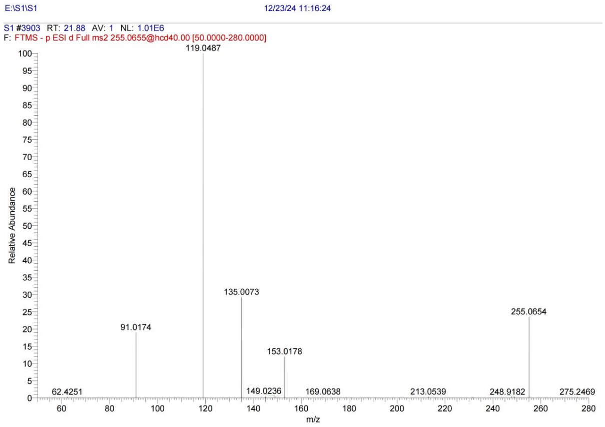

Figure S6. Liquiritigenin MS/MS fragments

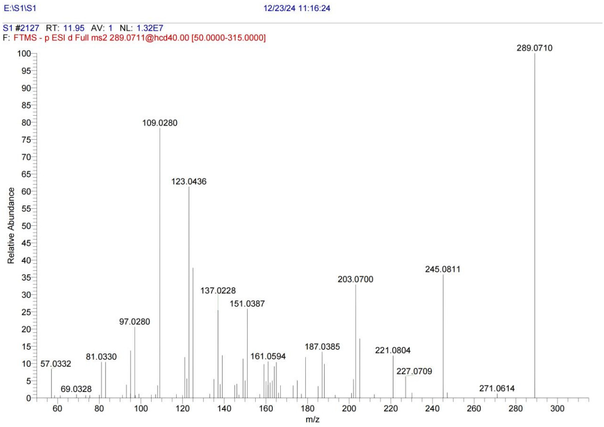

Figure S7. Epicatechin MS/MS fragments

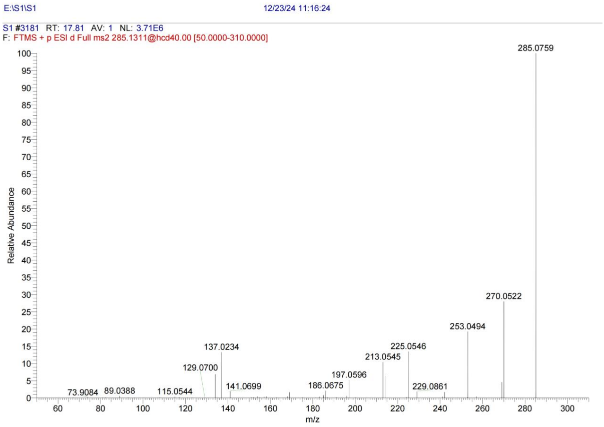

Figure S8. Wogonin MS/MS fragments

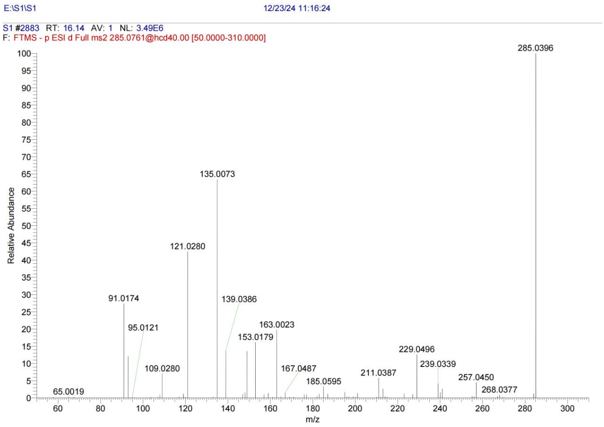

Figure S9. Fisetin MS/MS fragments

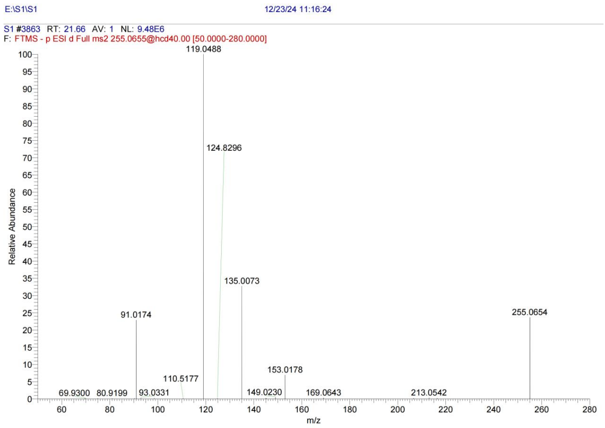

Figure S10. Isoliquiritigenin MS/MS fragments

4. *HepG2 experimental results*

Table S2. Inhibition of *Indigofera stachyodes* on HepG2 cells (n = 6)

Group	IR (%)
Control	0.00
S1	82.35±9.02****
S2	83.39±7.72****
S3	83.24±9.98****
S4	33.38±9.15****
S5	75.35±4.56****
S6	86.00±6.27****
S7	80.82±6.11****
S8	81.59±9.06****
S9	76.90±5.99****
S10	66.62±6.93****
S11	63.91±6.43****
S12	85.79±3.83****
S13	87.67±4.10****
S14	67.49±5.89****
S15	80.11±9.38****
S16	87.32±4.88****

Note: **** $P < 0.0001$, compare to control group.

5. *Principal component analysis (PCA)*

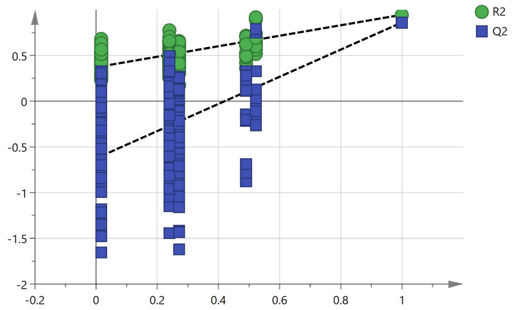

Figure S11. Permutation test plot for the PCA model

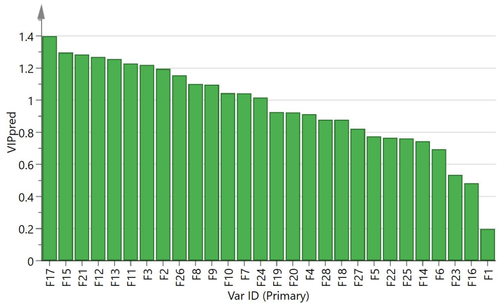

Figure S12. VIP scores plot for the PCA model
